# A German View of Intubation of the Larynx

**Published:** 1888-06

**Authors:** 


### Selections.

---

### *A GERMAN VIEW OF INTUBATION OF THE LARYNX.*

At the Seventeenth Congress of the German Society of Surgery, held in Berlin, April 4-7 ult., Thiersch, of Leipzig, gave his experience with O'Dwyer's method. He has performed intubation of the larynx in thirty-two cases of diphtheritic croup. In fourteen of these, he was obliged to remove the tube and perform tracheotomy, on account of attacks of suffocation. Only three patients got well after intubation, and without tracheotomy. He attributed these rather discouraging results to the exceptional gravity of croup in Leipzig. Intubation, Thiersch says, may be substituted for tracheotomy, in cases of croup where there is only a limited neo-membranous formation and no swelling of the vestibule of the larynx. On the other hand, when the epiglottis is tumefied, or when there is swelling of the mucous membrane below the vocal cords, or when there is an abundant membranous formation, tracheotomy is obligatory, and intubation can do no good.

In favorable cases, amelioration appears immediately after placing the O'Dwyer canula in the windpipe.

Some of the inconveniences of intubation (to say nothing of its difficulties) are as follows: Deglutition is badly performed; the child swallows the wrong way. This is due to the presence of a stiff, open tube in the larynx, and there is also danger of pneumonia by aspiration (Schluck pneumonie). On this point, he was disposed to lay some stress. Sometimes the child complains of pains, and indicates the seat; erosions are found in the trachea as the result of intubation, and may open the way to general infection. Sometimes the child expels the canula in a fit of coughing. Children treated by this method need constant watching, for they are in perpetual danger of œdema of the glottis, or of being choked by the membranes, and in such cases the presence of the physician is necessary to remove the tube and false membranes, and, it may be, perform tracheotomy.

Rehn, of Frankfort-on-the-Main, was of the same opinion as Thiersch on the subject of intubation. He had treated fourteen cases of croup by this method; ten of them died. The most of his patients swallowed with such difficulty after intubation, that he was obliged to feed them by the œsophageal sound or by lavements. In two instances, the canula was coughed up and swallowed by the patients, and afterwards passed by rectum.

Rose, of Berlin, said that he was not an advocate of intubation. He was an ardent tracheotomist, having performed the operation two thousand times, with a mortality of 71.6 per cent. This gave him a percentage of 28.4 of cures, to offset 21 per cent. of recoveries under intubation in America.—*Boston Medical and Surgical Journal.*

